# Evolutionary problems in centrosome and centriole biology

**DOI:** 10.1111/jeb.12620

**Published:** 2015-05-12

**Authors:** L. Ross, B. B. Normark

**Affiliations:** ^1^ Institute of Evolutionary BiologySchool of Biological SciencesUniversity of EdinburghEdinburghUK; ^2^ Department of Biology and Graduate Program in Organismic and Evolutionary BiologyUniversity of MassachusettsAmherstMAUSA

**Keywords:** axoneme, centriole, centrosome, inheritance, insect, paternal genome elimination, replicator, sperm

## Abstract

Centrosomes have been an enigma to evolutionary biologists. Either they have been the subject of ill‐founded speculation or they have been ignored. Here, we highlight evolutionary paradoxes and problems of centrosome and centriole evolution and seek to understand them in the light of recent advances in centrosome biology. Most evolutionary accounts of centrosome evolution have been based on the hypothesis that centrosomes are replicators, independent of the nucleus and cytoplasm. It is now clear, however, that this hypothesis is not tenable. Instead, centrosomes are formed *de novo* each cell division, with the presence of an old centrosome regulating, but not essential for, the assembly of a new one. Centrosomes are the microtubule‐organizing centres of cells. They can potentially affect sensory and motor characters (as the basal body of cilia), as well as the movements of chromosomes during cell division. This latter role does not seem essential, however, except in male meiosis, and the reasons for this remain unclear. Although the centrosome is absent in some taxa, when it is present, its structure is extraordinarily conserved: in most taxa across eukaryotes, it does not appear to evolve at all. And yet a few insect groups display spectacular hypertrophy of the centrioles. We discuss how this might relate to the unusual reproductive system found in these insects. Finally, we discuss why the fate of centrosomes in sperm and early embryos might differ between different groups of animals.

## Introduction

**Figure 1:**
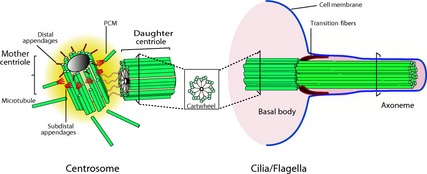
The dual role of centrioles in animals, either involved in cell division as part of the centrosome or in cell motility as part of the basal body of an axoneme. The figure on the left shows the structure of the centrosome consisting of two centrioles surrounded by the pericentriolar material (PCM). Each of the two centrioles consists of a ring of nine microtubule triplets, as shown in the insert in the middle section of the figure. The figure on the right shows how a centriole can attach to the cell membrane to act as a basal body and seed the growth of cilia and flagella. The microtubule skeleton within a cilium or flagellum is called the axoneme and has a similar nine‐fold structure as the centriole, but with 9 doublets instead of triplets. Reproduced from Bettencourt‐Dias (2013) with kind permission from the copyright holder.

The centriole is a eukaryote organelle involved in cell division, sensory reception, locomotion and embryogenesis. It may be found by itself or as part of a larger organelle – the centrosome (Fig. 1). Each centriole is a cylinder of microtubules, typically consisting of a ring of 27 microtubules (arranged as 9 triplets) surrounding 6 central microtubules (arranged as 2 triplets). The peculiar nine‐fold structure of the centriole is conserved across eukaryote kingdoms, but in a few groups, this structure becomes extremely variable and in other groups it is lost entirely. Why? Why should a structure be extraordinarily conserved yet dispensable, and why should that conservation – in only a few cases – break down? Evolutionary biologists have largely ignored these questions or else have sought to address them by invoking inaccurate models of centriole transmission (Normark, 2009). Here, we highlight a few paradoxes and enigmas of centriole and centrosome evolutions and seek to understand them in the light of recent advances in the centrosome biology. We focus primarily on the evolution and function of centrioles as part of centrosomes in animals, but will briefly discuss centriole evolution in other eukaryotes as well.

## The paradox of centrosome inheritance

Centrosomes are typically discussed as entities that replicate and have inheritance (Schatten *et al*., 1991; Callaini *et al*., 1999; Karr, 2001; Avidor‐Reiss *et al*., 2012; Pelletier & Yamashita, 2012), even though they lack nucleic acids and thus pose a challenge to conventional views about the mechanisms of heredity. A few evolutionary biologists have taken this challenge seriously and have discussed unorthodox alternative models of evolution invoking peculiar non‐Mendelian features of centrosome inheritance (Bermudes *et al*., 1987; Grafen, 1988; Normark, 2009). The paradox of centrosome inheritance is, fortunately, easy to resolve. There is sufficient evidence to demonstrate convincingly that there is no such thing as ‘centrosome duplication’ or ‘centrosome inheritance’ despite the near‐universal use of these terms. Here, we briefly review key advances in centrosome biology and how they falsify the notion of centrosome ‘inheritance’.

During each cell division (both meiosis and mitosis), a new centrosome forms in the vicinity of the old centrosome, a process referred to as canonical duplication. This observation led to the assumption that the old ‘mother’ centrosome serves as a template for its ‘daughter’ and led to speculation that the centrosome is a true replicator that can evolve independently of the nuclear genome (e.g. Grafen, 1988). Throughout the 20th century, there was much debate about the status of the centrosome as a replicator and especially how the template information is inherited (Andersen, 1999). At first, it was assumed that centrosomes (and centrioles), like mitochondria and chloroplasts, contained DNA. After a number of erroneous claims, this notion was refuted (Johnson & Rosenbaum, 1990; Dirksen, 2012). However, even without DNA, it was still possible that centrosomes served as template through other unknown means (Grafen, 1988). The best way to test whether one centrosome serves as the template for another would be to study cases of intraspecific variation in centrosome or centriole structure. Such variation exists in a few groups of animals, including sciarid flies. A study of one such fly in the genus *Sciara* showed that unusual ‘giant’ centrioles restricted to the germ line formed next to ‘old’ centrioles with conventional 9 + 2 structure (Phillips, 1967). These results demonstrate that the old centrosome does not serve as a template for replication in this case. More recent experiments show that in cells where the centrosomes are experimentally removed, new centrosomes can originate *de novo* (La Terra *et al*., 2005), again clearly demonstrating that centrosomes in this case originate by a mechanism other than replication. It is conceivable that there are two different modes of centrosome origination: centrosomes may replicate, or they may be assembled *de novo* without a template. But it is more parsimonious to hypothesize that centrosomes are always assembled *de novo* without reference to a template, even though they are often assembled in the proximity of an existing centrosome, creating the illusion of replication. This is in fact the prevailing model of centrosome assembly, although most authors confusingly persist in referring to centrosome replication and inheritance and in using ‘*de novo’* to refer only to the case of centrosome assembly in the absence of a pre‐existing centrosome. Thus, the ‘canonical pathway’ refers to centrosome assembly in the vicinity of an existing centrosome, and the ‘*de novo* pathway’ refers to centrosome assembly in the absence of any centrosome. The presence of a centriole suppresses the *de novo* pathway (La Terra *et al*., 2005; Tsou & Stearns, 2006) such that the majority of centriole assembly across organisms and cell types occurs through the canonical pathway. Both the canonical and *de novo* pathways are controlled by the kinase SAK/PLK4 and involve the proteins SAS‐4 and SAS‐6 (Rodrigues‐Martins *et al*., 2007). SAS‐6 functions by forming a cartwheel structure acting as a scaffold for the formation of the new centrioles (Kitagawa *et al*., 2011; Bornens, 2012). For more detailed accounts of the molecular mechanisms of centrosome assembly, see Nigg and Stearns (2011), Gönczy (2012) and Bettencourt‐Dias (2013). One explanation for why canonical formation of the centrosomes is more common than *de novo* formation is that the formation of too many centrosomes reduces genomic stability, as often seen in cancerous cells (Tsou & Stearns, 2006). The reason for the usual vicinity between mother and daughter centrioles could be that centrosomes occur in favourable environments for centrosome assembly, perhaps because of the local enrichment of microtubules or other centrosomal components (Kitagawa *et al*., 2011). It has recently been discovered that the daughter centriole is attached to the mother centriole by a stalk which initiates the assembly of the cartwheel structure (Fırat‐Karalar & Stearns, 2014). However, although a close proximity or even attachment between mother and daughter centrioles aids centrosome assembly, there is nothing to suggest that the mother centriole serves as a template in this process.

Thus, the centrosome is not a replicator but instead a phenotype whose structure is determined by the nuclear genome: the centriole neither acts mechanistically as a template, nor is there evidence for traits inherited via the centriole separately from the nuclear genome. Although it is conventional to speak of centrosome duplication and centrosome inheritance, these are confusing misnomers. It is better to speak of ‘centrosome assembly’ and to distinguish between the canonical and *de novo* pathways to centrosome assembly, which differ in whether centrosome assembly is centrosome induced or not (Avidor‐Reiss *et al*., 2012).

## Ultraconserved yet dispensable

The extremely conserved nine‐fold structure of centrioles, and their prominent role in eukaryotic cells across eukaryote phyla, suggests not only that they are an essential organelle, but also that their precise structure is essential and that variation in this structure either does not occur or is necessarily deleterious. And yet, in a number of major groups of eukaryotes, centrioles have been entirely lost (Azimzadeh & Bornens, 2005; Debec *et al*., 2010; Bettencourt‐Dias, 2013). This is a somewhat more difficult paradox to understand, but it seems to hinge on the dual function of centrioles – in cilia and in cell replication. Centrioles seem to be utterly essential for cilia but much less important for cell replication (Debec *et al*., 2010). For example, *Drosophila* embryos with ablated centrosomes develop normally until the point in late development when they need – and are unable – to produce ciliated cells (Martinez‐Campos *et al*., 2004). And yet in spite of the fact that centrioles are not required for cell replication, animals usually do employ them for this purpose. Here, we review recent information on centriole function and functional necessity and what this can tell us about centrioles’ evolution and taxonomic distribution.

Centrioles evolved early in the history of eukaryotes and with a few exceptions are found in all major eukaryote clades (Azimzadeh & Bornens, 2005; Debec *et al*., 2010; Hodges *et al*., 2010; Carvalho‐Santos *et al*., 2011). Centrosomes evolved much later and are restricted to animals and some fungi, where they serve as the microtubules‐organizing centre of dividing cells (Hodges *et al*., 2010; Carvalho‐Santos *et al*., 2011). Other eukaryotes do have microtubule‐organizing centres, but these lack centrioles (Azimzadeh & Bornens, 2005). So centrioles became part of the cell division machinery relatively late in the evolution of eukaryotes. This suggests that centrioles first evolved to fulfil a different function, most likely as the basal body of the axoneme (Fig. 1) within cilia and flagella, thereby providing cells with their mobility (Debec *et al*., 2010). Support for this comes from those groups of organisms that completely lack cilia (e.g. higher plants and red algae) which have also lost centrioles and from many lower plants that lack centrioles in most cells, but form centrioles in motile spermatozoa (Marshall, 2009). In animals, which use centrosomes to organize their microtubule cytoskeleton, the centrosome has many other functions apart from its role in cell division and cell motility, which include signalling, adhesion, the coordination of protein trafficking by the microtubule cytoskeleton and the establishment of polarity (Bettencourt‐Dias, 2013). However, these other functions appear to be nonessential, as the centrosome often is absent or inactive in fully differentiated cells that no longer divide. In fact, even its role in cell division only appears to be essential in certain tissues (Rodrigues‐Martins *et al*., 2008; Bettencourt‐Dias, 2013). Mouse embryos lack a centrosome until the 64‐cell stage (Courtois *et al*., 2012). Recent data from *Drosophila* mutants lacking centrioles suggest that in fact, the centrosome's role in cell division is only truly essential for male (but not female) meiosis (Rodrigues‐Martins *et al*., 2008). Finally, it turns out that even in animals, the centrosome can be lost secondarily. The flatworm *Planaria* does not have a centrosome, even though it does use centrioles to construct cilia (Bettencourt‐Dias, 2013), corroborating motility as the only function for which a centriole is apparently indispensable.

In short, the centriole's first and most indispensable role is to provide the axoneme of cilia and flagella, thereby providing mobility to cells. In animals, centrioles are now also involved in a range of other functions, most importantly cell division, where they are not essential except during male meiosis. Why the centrosome appears particularly indispensable for male meiosis remains a question open to debate.

## Ultraconserved or ultravariable

**Figure 2:**
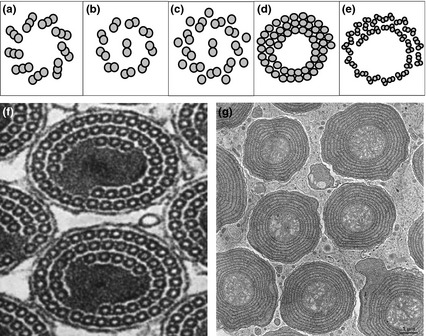
Centriole/axoneme structures. (a) The structure of a typical centriole consists of a ring of nine microtubule triplets with two triplets in the middle (often referred to as the 9 + 2 structure). This structure is conserved in the vast majority of taxa that possess centrioles, with only minor variations. (b) The nine‐fold symmetric pattern is also present in the axoneme, the centriole‐derived cilia found, for example, in most sperm cells, although the axoneme consists of 9 doublets instead of the 9 triplets found in the centriole. (c) The alternative axoneme structure found in most insects (9 + 9 + 2) where the 9 doublets are surrounded by a ring of 9 singlets. Alternative structures without nine‐fold symmetry have evolved just a handful of times, mostly in insects (Riparbelli *et al*., 2010), with the most highly aberrant axoneme structures in (d) scale insects and (e) cecidomyiid flies. The photographs show axonemes of (f) an armored scale insect (reprinted with permission from Robison, 1972) and (g) a cecidomyiid gall midge (courtesy Romano Dallai).

**Figure 3:**
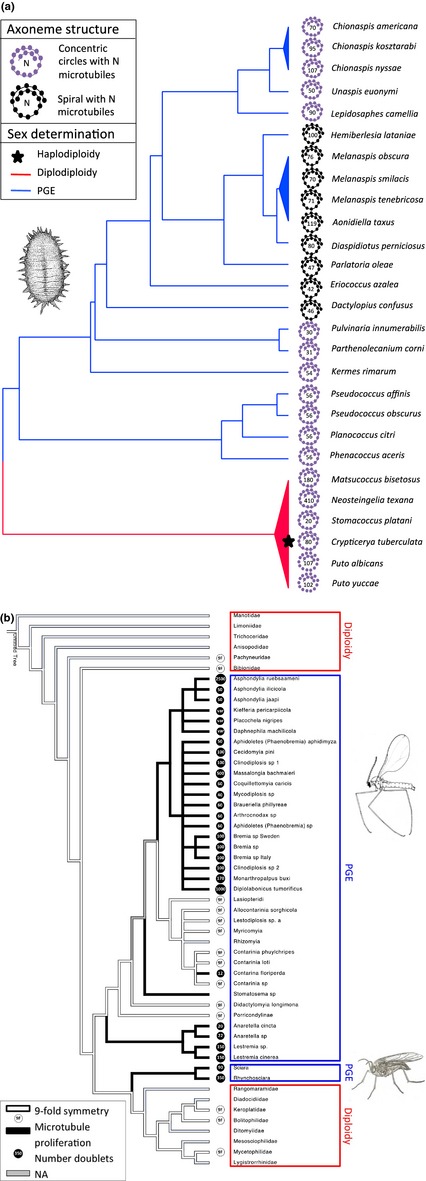
Centriole/axoneme structure in scale insects and sciarid and cecidomyiid flies. (a) The evolution of axoneme structure and PGE among scale insects. Phylogeny based on Ross *et al*. 2013. There are two types of axonemes – spirals and concentric circles – each of which varies in the number of microtubules of which it consists. Data on axoneme structure from Robison (1990); data on sex determination systems from Ross *et al*. (2012, 2013). All scale insects display unusual axoneme structures, in contrast to their sister group the aphids, which have axonemes with the conventional nine‐fold structure (Bào *et al*., 1997). (b) The evolution of unusual centriole/axoneme structure among flies (Diptera). Phylogeny based on Amorim & Rindal (2007) and (within Cecidomyiidae) Jamieson *et al*. (1999). Flies have two different types of axoneme: the standard insect type with nine‐fold radius and a spiral with varying numbers of microtubules. Data from Jamieson *et al*. (1999) and Dallai (2014). Here, we show the phylogenetic distribution of the different axoneme types as well as different sex determining systems (PGE vs. diploidy).

Although centriole structure is conserved across the great majority of eukaryotes, there are a few groups, in insects and Heliozoa (Mikrjukov & Patterson 2001; Riparbelli *et al*., 2010), where this conservation is lost, and centriole structure becomes extremely variable, at least in the male germ line and axoneme (Normark, 2009). Typically, there is a proliferation of microtubules, with different numbers in closely related species (Figs 2 and 3a,b). This is perhaps a deeper enigma than the others, but one possible hint is a repeated association between centriole novelties and paternal genome elimination (PGE), a reproductive system in which all chromosomes of paternal origin are eliminated from the male germ line.

In both scale insects (Hemiptera: Coccoidea) and fungus gnats (Diptera), which share the features of unusual centrioles and PGE, centrioles have lost their nine‐fold symmetry and become very large with up to thousands of microtubules (Figs 2 and 3a,b) (Phillips, 1967; Robison, 1990; Callaini *et al*., 1999; Paoli *et al*., 2015). These ‘giant’ centrioles are not found in every cell, though. In fact, they appear restricted to the male germ line, where they give rise to axonemes of similarly remarkable structure (Phillips, 1967). Why would we expect species with PGE to display unusual centrosomal structures? In principle, any of the phenotypes affected by the centrosome might be important under PGE, but because the unusual structures are restricted to the male germ line, we believe that selection on either male meiosis or sperm performance is most likely.

Sperm function and motility have previously been shown to be correlated with axoneme structure across invertebrates (Carvalho‐Santos *et al*., 2011). PGE imposes unique selection on the mature sperm and its axoneme: under PGE, some eggs are destined to be male and thus to eliminate the genome of any sperm entering them (Normark, 2009; Shuker *et al*., 2009; Featherston *et al*., 2013). There might be strong selection for sperm to be able to detect this cue and thus strong selection for a sensory capability for sperm. There might also be strong selection for sperm to avoid such eggs and seek female‐determined eggs, which might select for greater sperm motility. Another aspect of PGE that might affect sperm motility is that all sperm of a PGE male are genetically identical. This obviates competition between the gametes of an individual male and might therefore reduce the strength of selection on the swimming ability of individual sperm, especially under monogamy.

Possibly more relevant to centrosome hypertrophy under PGE are the effects of the centrosome on the movement of chromosomes during male meiosis, in which only the maternal chromosomes are included in the sperm, whereas the paternal chromosomes disintegrate. Let us hypothesize (following e.g. Herrick & Seger, 1999) that PGE results from genomic conflict within males. Specifically, a male's maternal chromosomes, expressed in his germ line, eliminate his paternal chromosomes to enhance their own transmission rate, doubling that rate from 50% to 100%. But how can the maternal chromosomes accomplish this? In both Diptera and scale insects, the mechanism of PGE involves the formation, during male meiosis, of a monopolar spindle (Bongiorni *et al*., 2004). The monopolar spindle pulls the maternal chromosomes to one side, whereas the paternal chromosomes are left behind. This is in contrast to a normal bipolar spindle, in which each of two poles pulls half of the chromosomes to itself. According to Bongiorni *et al*. (2004), ‘the monopolar spindle could originate from a lack of canonical centrosome assembly in secondary spermatocytes’. Let us therefore further hypothesize that the maternal chromosomes express some gene product that initiates PGE by interfering with centrosome assembly. In this scenario, centrosome novelties in the male germ line could have arisen as a paternal‐gene response to these maternally expressed suppressors of centrosome assembly: a chemically or structurally novel centrosome component might not be recognized by the suppressor and thus might escape suppression. Centrosome proteins expressed from paternal chromosomes would be under selection to promote centrosome assembly in spite of suppressors and might evolve novel features for this purpose – indeed, there is the potential for an evolutionary arms race between maternally expressed suppressors of centrosome assembly and paternally expressed centrosome components.

Although the association between unusual centrioles and PGE is tantalizing, the two phenomena could of course be unrelated and co‐occur by chance. The co‐occurrence has evolved 2–3 times independently (depending whether sciarid and cecidomyiid flies constitute independent origins of PGE; see Fig. 3b), but in scale insects, the association is not perfect, as unusual centriole structures appear to have evolved prior to the evolution of PGE (Fig. 3a). If PGE imposes selection on centriole or axoneme structure, then we might expect to see unusual structures in other taxa with PGE. PGE is found in thousands of species across insects, springtails and mites, and has evolved at least seven times (Gardner & Ross, 2014). Species belonging to three of these origins are discussed above. For the remaining four, the evidence is mixed, and often there is no information available. In the springtails with PGE, the axoneme structure is well studied and appears to adhere to the classic 9 + 9 + 2 structure typical of most insects and their close relatives (Dallai, 2014). Interestingly however, the Protura, the closest out‐group of springtails, does show an unusual axoneme structure (14 + 0) (Dallai *et al*., 2010). Another taxonomic group with PGE that shows unusual sperm axonemes are the sucking lice (Anoplura), where the sperm flagellum has not one but two axonemes, derived from two separate centrioles (Baccetti *et al*., 1969). All lice show a highly aberrant male meiosis, but PGE has only been confirmed in one species, the human body louse (McMeniman & Barker, 2005). The only other occurrence of a sperm flagellum containing two or more axonemes is found in the haplodiploid thrips (Thysanoptera) (Baccetti *et al*., 1969, Paccagnini *et al*., 2007). For the remaining two origins of PGE, one in the coffee borer beetle and one in mites, there is no direct information on centrosome or axoneme structure, but centrosomes and axonemes are absent from the male germ line of all mites studied to date (Florek & Witalinski, 2010). Although scale insects, Sciaridae, and Cecidomyiidae display the largest variety of unusual centriole structures by far, a few other insect groups have variable centrioles that lack nine‐fold symmetry. Examples include the proturans (Dallai *et al*., 2010) and some trichopterans (Dallai *et al*., 1995). The reproductive biology of both groups has been poorly studied, and it would be of great interest to see whether PGE might be found in these groups upon further investigation.

Besides PGE, there are a number of other genetic systems that involve genomic exclusion (Burt & Trivers, 2006), and here, we consider the role and structure of the centrosome in these systems. Under gynogenesis, females reproduce clonally, but mate with either conspecific males or males from a closely related species to activate their eggs. The requirement for sperm is often attributed to the need for a paternally derived centrosome (Neaves & Baumann, 2011). Gynogenesis intrinsically involves sexual antagonism because males derive no genetic benefit from mating with gynogenetic females and are under selection to avoid doing so. But this selection may be weak, especially if gynogenetic females are uncommon (compared to the males’ sexual female conspecifics) and if the cost of mating is low. Selection on males to evolve centrosome novelties to overcome such elimination is probably also weak, especially as this might interfere with the viability of normal (nonhybrid) zygotes. Hybridogenesis is similar to gynogenesis, except that the male's genome is incorporated into the F1 offspring's somatic genome, but is then eliminated from the offspring's germ line, such that the offspring (always female) produce eggs containing only the haploid genome they received from their mother. Thus, males have no F2 progeny. Again, this is a system with intrinsic sexual antagonism, but again, it tends to occur in situations in which selection on males to resist it is relatively weak (e.g. when hybridogens are uncommon compared to the males’ sexual conspecific females).

A system with greater potential for sexually antagonistic centrosome evolution is androgenesis. Here, the sperm genome completely replaces the egg genome, giving rise to effectively clonal reproduction via sperm. Although this type of reproduction is found in a number of taxonomic groups including a cypress, a stick insect and a few species of ants, the mechanism has only been studied in detail in a few species of *Corbicula* clams (Pigneur *et al*., 2012). *Corbicula* eggs are arrested in meiosis, and meiosis is only completed upon fertilization. In androgenetic species, the two maternal centrosomes attach to the egg cortex and pull themselves and all maternal chromosomes into 2 polar bodies, which are ejected from the egg (Komaru *et al*., 2000). The sperm, on the other hand, is diploid and biflagellate, containing two axonemes with the typical 9 + 2 pattern (Komaru & Konischi, 1996). The presence of functional maternally derived centrosomes in *Corbicula* eggs is itself unusual; in the eggs of most animals, the centrosomes disintegrate in early oogenesis (Manandhar *et al*., 2005). This system seems more conducive to the evolution of sexually antagonistic centrosome features than either hybridogenesis or gynogenesis, because there is obligate conspecific mating. Thus, females’ ‘antagonists’ (androgenetic males) are ubiquitous rather than being uncommon, which exerts stronger selection. And thus, there is no ‘normal sexual development’ with which any centrosome novelties might interfere. Nonetheless, apart from the unusual sperm morphology with two axonemes, there does not seem to be any evidence for structural abnormalities of the centrosome or axonemes themselves. Androgenesis, hybridogenesis and gynogenesis are all effectively asexual systems, and – perhaps for this reason – of recent origin. PGE is effectively sexual and much more ancient. This may help to explain why elaborate centrosome anomalies have evolved in the context of PGE and not in the context of these other systems of genome elimination. PGE also mechanistically depends upon the existence of genomic imprinting, a phenomenon that provides a wide scope for genomic conflict (Burt & Trivers, 2006). Except possibly for hybridogenesis, the other systems of genome elimination do not require genomic imprinting, and if these occur in groups that lack genomic imprinting, the range of mechanisms available for the evolution of centrosome anomalies may be drastically reduced.

## What induces centrosome assembly in the zygote, and why does it vary?

Typically, a sperm‐derived centriole induces centrosome assembly in the zygote, but this is not always the case (Schatten *et al*., 1991; Callaini *et al*., 1999; Bornens, 2012). During both male gametogenesis and female gametogenesis, the centrosome partly disintegrates (Manandhar *et al*., 2005). This process leads to the complete loss of both centrioles during oogenesis in all species studied to date (except in *Corbicula* clams as discussed previously). However, the process is more complex and variable during spermatogenesis, in which, depending on the taxonomic group, one, both or neither of the centrioles may be lost (Manandhar *et al*., 2005). This has important implications for centrosome formation during fertilization. In species in which sperm introduce two centrioles, both of these induce the formation of a new centriole and these four centrioles form the basis of the two centrosomes in the zygote. In other groups, including primates, one of the centrioles is highly degraded and presumably the single nondegraded centriole induces the formation of all four centrioles in the zygote. Finally, in some groups, such as rodents, some snails and stick insects, both centrioles are lost (Manandhar *et al*., 2005). As a result, the zygote does not receive a centriole upon fertilization and all new centrioles are produced *de novo,* presumably from maternally derived components.

It is currently unclear why centrioles are lost from oocytes. Prevention of spontaneous parthenogenesis is posed as an explanation why the centrosome is usually paternally derived (Manandhar *et al*., 2005). Indeed, the stick insects, one of the few groups that lack paternally derived centrosomes, frequently transition to parthenogenesis (Schwander *et al*., 2011). And fish and amphibians, where the paternally derived centriole appears essential, seem only able to evolve asexual reproduction through sperm‐dependent parthenogenesis (gynogenesis or hybridogenesis). On the other hand, most species seem able to form centrosomes *de novo* from maternal proteins in the absence of a paternal copy. And many parthenogenetic insects (other than stick insects) have sexually reproducing conspecifics or sister species that do receive a paternal centrosome (de Saint Phalle & Sullivan, 1998; Tram & Sullivan, 2000; Ferree *et al*., 2006). Another possible explanation is that having both parents contribute, a centrosome might disrupt early embryogenesis (Manandhar *et al*., 2005). Several lines of evidence corroborate the notion that having too many centrioles or centrosomes can lead to pathology (Nigg, 2002; Snook *et al*., 2011). Therefore, as a sperm cell needs a centriole to form an axoneme, while an oocyte does not do anything that requires a centriole, it should not be surprising that sperm often contribute the first centriole to the zygote. However, there appears to be intraspecific variation in how well certain organisms can tolerate superfluous centrioles. A recent review of polyspermy in animals shows that in some species, early embryogenesis is severely disrupted when multiple sperm, each carrying a centriole, enter an oocyte, whereas in other species, these superfluous centrioles simply degrade without causing any negative effects (Snook *et al*., 2011). Hopefully with a better and taxonomically broader understanding of the role of centrioles in sperm and early embryogenesis, it should be possible to test these hypotheses in a comparative framework – for example, whether taxa with *de novo* assembly of centrioles are more likely to evolve parthenogenesis, whereas those with male‐derived centrioles are more likely to evolve gynogenesis, or whether species with nonmotile sperm (Werner & Simmons, 2008) that lack an axoneme are more likely to lose sperm‐derived centrioles.

The available comparative evidence suggests that indeed in the majority of cases, the sperm introduces a centriole into the oocyte. This has been confirmed, for example, for most mammals, including humans, pigs, porcupines, cats and cows (Manandhar *et al*., 2005). Notable exceptions are the rodents and their sister clade the rabbits, in which oocytes do not tolerate sperm‐derived centrioles and instead new centrosomes are formed *de novo* (Szollosi *et al*., 1972; Schatten *et al*., 1991), a trait most likely evolved just once in their common ancestor. *De novo* assembly of centrosomes is also observed in a large variety of parthenogenetically reproducing invertebrates (Callaini *et al*., 1999), including hymenopterans, flies, aphids and *Daphnia*. The closest sexual relatives of these parthenogenetic lineages usually depend on paternally contributed centrioles (de Saint Phalle & Sullivan, 1998; Hiruta & Tochinai, 2012), with the exception of stick insects, in which *de novo* formation is observed in both sexual and parthenogenetic species (Marescalchi *et al*., 2002). Finally, an example of just how variable transmission patterns can be comes from haplodiploid species, where the parental origin of the centrosome is dependent on offspring sex: females developing from fertilized eggs receive the paternal centrosome, whereas males developing from unfertilized eggs assemble their centrosome *de novo* (Tram & Sullivan, 2000).

Although ‘centrosome inheritance’ is a misnomer, centrioles do show paternal ‘transmission’ from sperm to zygote in many taxa. Although this centriole does not serve as a template for further centriole assembly, it does play a role in the organization of early embryogenesis and is important for bringing the male and female pronuclei together after fertilization (Manandhar *et al*., 2005). The period before the fusion of the pronuclei is important for the reorganization of epigenetic marks, and it is thought that at least in mammals, many parent‐of‐origin‐specific epigenetic marks are established during this period (Kelsey & Feil, 2013). It could therefore be significant if at least part of the process were subject to substantial paternal effects, although this remains speculative until we have a better understanding of the role of the paternal centriole in early development.

## Conclusion

(1) Centrosomes are not true replicators, and phrases such as ‘centrosome inheritance’ and ‘centrosome transmission’ should be avoided to minimize confusion. (2) Canonical (centrosome induced) centrosome formation may enable tighter control of centrosome number than *de novo* centrosome formation, which may reduce the chance of supernumerary centrosomes that can disrupt the cell cycle and lead to pathology. (3) The giant centrioles in some flies and scale insects may have evolved as a result of maternal–paternal conflict over the elimination of paternal chromosomes from the male germ line. (4) Reliance on a sperm‐derived centrosome in the zygote might have evolved to streamline embryo activation upon fertilization and avoid spontaneous oocyte activation. Research into centrosome development and function across a wide diversity of organisms, in particular in those lineages that have independen‐tly evolved unusual centrosomes, is needed to resolve the remaining evolutionary problems in centrosome biology.
